# Supplementary material for: The Construction and Exploration of a Comprehensive MicroRNA Centered Regulatory Network in Foxtail Millet (Setaria italica L.)
Source: Front Plant Sci. 2022 May 6;13:848474. doi: 10.3389/fpls.2022.848474 (PMC9121102; doi:10.3389/fpls.2022.848474)
Supplement: Supplementary file 1 [file Data_Sheet_1.zip › Sit-miRN3020_Seita.9G517700.1_1680_TPlot.pdf]

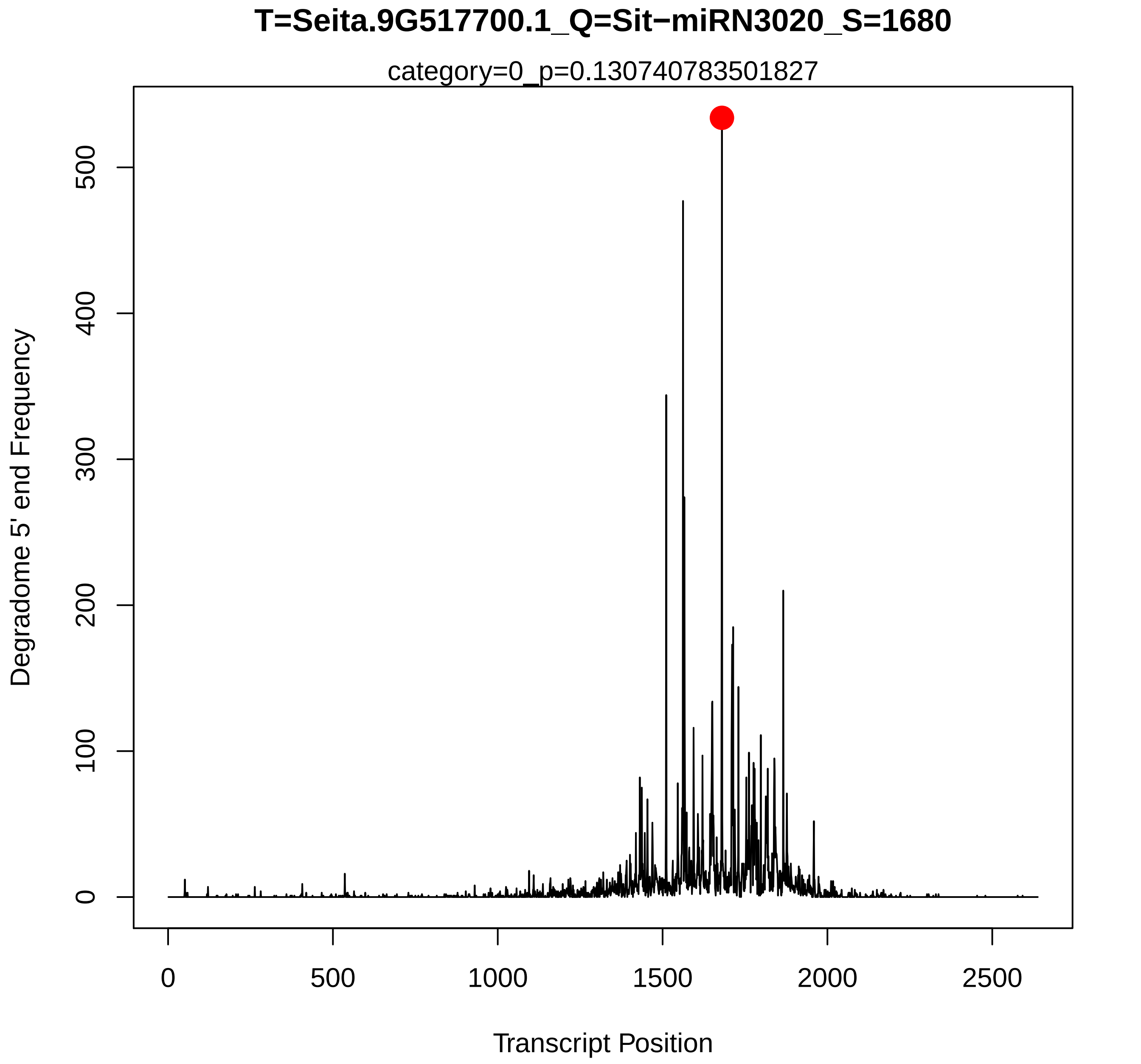

T=Seita.9G517700.1_Q=Sit−miRN3020_S=1680
category=0_p=0.130740783501827
Degradome 5' end Frequency
0
100
200
300
400
500
0
500
1000
1500
2000
2500
Transcript Position
